# *MIR376A* Is a Regulator of Starvation-Induced Autophagy

**DOI:** 10.1371/journal.pone.0082556

**Published:** 2013-12-16

**Authors:** Gozde Korkmaz, Kumsal Ayse Tekirdag, Deniz Gulfem Ozturk, Ali Kosar, Osman Ugur Sezerman, Devrim Gozuacik

**Affiliations:** Faculty of Engineering and Natural Sciences, Sabanci University, Istanbul, Turkey; French National Center for Scientific Research - Institut de biologie moléculaire et cellulaire, France

## Abstract

**Background:**

Autophagy is a vesicular trafficking process responsible for the degradation of long-lived, misfolded or abnormal proteins, as well as damaged or surplus organelles. Abnormalities of the autophagic activity may result in the accumulation of protein aggregates, organelle dysfunction, and autophagy disorders were associated with various diseases. Hence, mechanisms of autophagy regulation are under exploration.

**Methods:**

Over-expression of hsa-miR-376a1 (shortly *MIR376A*) was performed to evaluate its effects on autophagy. Autophagy-related targets of the miRNA were predicted using Microcosm Targets and MIRanda bioinformatics tools and experimentally validated. Endogenous miRNA was blocked using antagomirs and the effects on target expression and autophagy were analyzed. Luciferase tests were performed to confirm that 3′ UTR sequences in target genes were functional. Differential expression of *MIR376A* and the related *MIR376B* was compared using TaqMan quantitative PCR.

**Results:**

Here, we demonstrated that, a microRNA (miRNA) from the *DLK1/GTL2* gene cluster, *MIR376A*, played an important role in autophagy regulation. We showed that, amino acid and serum starvation-induced autophagy was blocked by *MIR376A* overexpression in MCF-7 and Huh7 cells. *MIR376A* shared the same seed sequence and had overlapping targets with *MIR376B*, and similarly blocked the expression of key autophagy proteins ATG4C and BECN1 (Beclin 1). Indeed, 3′ UTR sequences in the mRNA of these autophagy proteins were responsive to *MIR376A* in luciferase assays. Antagomir tests showed that, endogenous *MIR376A* was participating to the control of *ATG4C* and *BECN1* transcript and protein levels. Moreover, blockage of endogenous *MIR376A* accelerated starvation-induced autophagic activity. Interestingly, *MIR376A* and *MIR376B* levels were increased with different kinetics in response to starvation stress and tissue-specific level differences were also observed, pointing out to an overlapping but miRNA-specific biological role.

**Conclusions:**

Our findings underline the importance of miRNAs encoded by the *DLK1/GTL2* gene cluster in stress-response control mechanisms, and introduce *MIR376A* as a new regulator of autophagy.

## Introduction

Two major degradation pathways, namely macroautophagy (autophagy herein) and the ubiquitin-proteasome system, are operational in the maintenance of cellular homeostasis. Functional at a basal level for long-lived protein degradation and organelle turnover under normal conditions, autophagy is rapidly upregulated in response to both extracellular (e.g. nutrient starvation, hypoxia) and intracellular (e.g. accumulation of unfolded proteins, damaged organelles, pathogens) stress factors.[1] Concerted action of several protein complexes formed by at least 32 different autophagy (ATG) proteins result in the formation of double- or multi-membrane vesicles called autophagic vesicles or autophagosomes.[2] These vesicles enwrap cargo molecules and carry them to lysosomes for degradation, resulting in the recycling of their constituents for reuse by the cell.[3], [4]

Protein complexes playing a role in autophagosome formation are numerous. A key event is the accumulation of a modified lipid molecule, phosphoinositol 3-phosphate on the ER and mitochondrial membranes, marking the autophagic vesicle nucleation centers.[2] A phosphoinositol 3-kinase, VPS34, is responsible for the conversion of membrane associated inositol lipids into phosphoinositol 3-phosphate (PI3-P). BECN1 was discovered as a master regulator of the VPS34 activity and autophagosome formation.[2] Autophagic vesicle membrane elongation, growth and closure occur through the action of two ubiqituination-like protein conjugation systems.[5] The first system is rather regulatory, resulting in the covalent conjugation of a ubiquitin-like protein ATG12 to ATG5, and in the eventual formation of a larger complex including the ATG16 protein. The ATG12-5-16 complex serves as a E3 ubiquitin ligase-like enzyme for the second reaction involving covalent attachment of a lipid, phosphatidylethanolamine (PE), to a carboxy-terminal (C-ter) glycine residue of the autophagy-related MAP1LC3 (or simply LC3) protein.[6] To expose the key glycine residue for conjugation, prior C-ter cleavage of pro-LC3 by ATG4 proteins is required. Lipid conjugated LC3 is necessary for the elongation of autophagic membranes and completion of the vesicles.[7] Indeed, cells lacking one of the conjugation reaction components were shown to harbor autophagy defects.[8]

Recent studies introduced microRNAs (miRNAs) as novel regulators of autophagy.[9], [10], [11] miRNAs are small non-coding RNAs serving as negative regulators of gene expression.[12] By base pairing with sequences found mainly in the 3′ untranslated region (3′UTR) of specific mRNAs, miRNAs lead to mRNA instability and/or translation inhibition resulting in a decrease in target gene expression.[13] A single miRNA may target tens to hundreds of mRNAs, hence may co-regulate and coordinate a number of cellular proteins and pathways at once.[14] So far, only a handful of miRNAs were shown to directly affect the autophagic activity. Among them, *MIR376B* was introduced as a new regulator of starvation and mTOR-inhibition-related autophagy.[10] *MIR376B* blocked autophagy by affecting the expression of two key autophagy proteins, namely ATG4C and BECN1. *MIR376B* belongs to a miRNA gene family encoded from a gene cluster region in the human chromosome 14q32, called the *DLK1/GTL2* region.[15], [16], [17] Therefore, we wondered whether other miRNAs from the same region could play a role in autophagy regulation.

Here, we report that another miRNA from the *DLK1/GTL2* region, namely hsa-miR-376a1 (hereafter *MIR376A*) containing a seed sequence similar to that of *MIR376B*, is a novel regulator of autophagy. Overexpression of *MIR376A* attenuated starvation-induced autophagic activity and did so by modulating cellular ATG4C and BECN1 mRNA and protein levels. We showed that, miRNA response elements (MRE) in the 3′UTR region of these genes were direct targets of *MIR376A*. Importantly, antagomir-mediated suppression of endogenous *MIR376A* levels led to an increase in ATG4C and BECN1 expression and resulted in autophagy stimulation. Our findings underline the importance of miRNAs coded by the *DLK1/GTL2* genomic region in physiological regulation and control of the autophagic activity.

## Results

### *MIR376A* overexpression blocked autophagy

**Figure 1:**
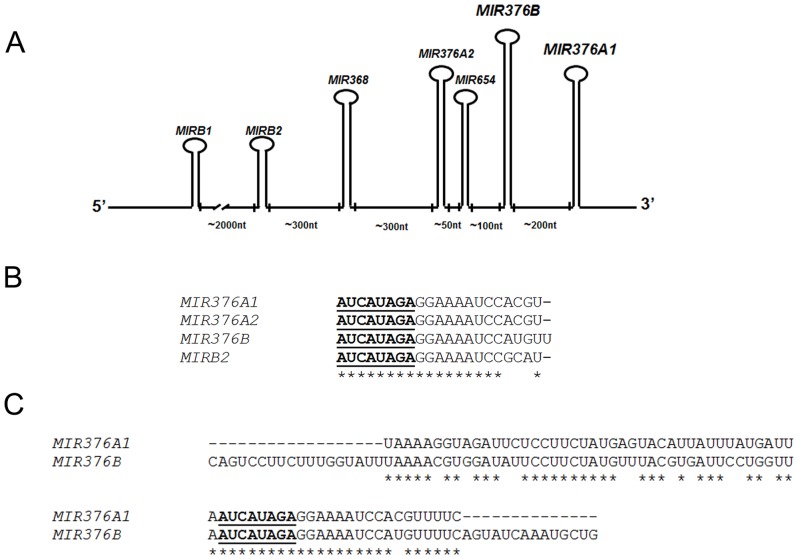
*MIR376* family miRNAs in the *DLKI/GTL2* gene cluster. (A) Individual miRNA genes were indicated. Sizes of spacer sequences between miRNA genes were marked as nucleotides (nt). (B) Comparison of mature miRNA sequences with identical “seed sequences” (bold, underlined). Identical nucleotides were indicated by asterix (*). ClustalW tool was used for the analysis. (C) ClustalW comparison of the stem-loop sequences of *MIR376A* and *MIR376B*. Seed sequences were marked as bold and underlined.

In an unbiased screen, we discovered several miRNAs, including *MIR376B* and *MIR181A* as inhibitors of starvation-induced autophagy [10], [11] *MIR376B* is encoded from a gene region called, *DLK1/GTL2* containing several miRNA genes (Figure 1A). Although the nucleotide sequences of at least seven miRNA genes found in this region differed considerably, the seed sequences (around 8 nucleotide long miRNA core sequences responsible for target mRNA recognition) of *MIR376A1, MIR376A2* and *MIRB2* were identical to that of the *MIR376B* (Figure 1B and C). Since in our screen, *MIR376A1* was also a hit, we wondered whether this miRNA would regulate autophagy.

**Figure 2:**
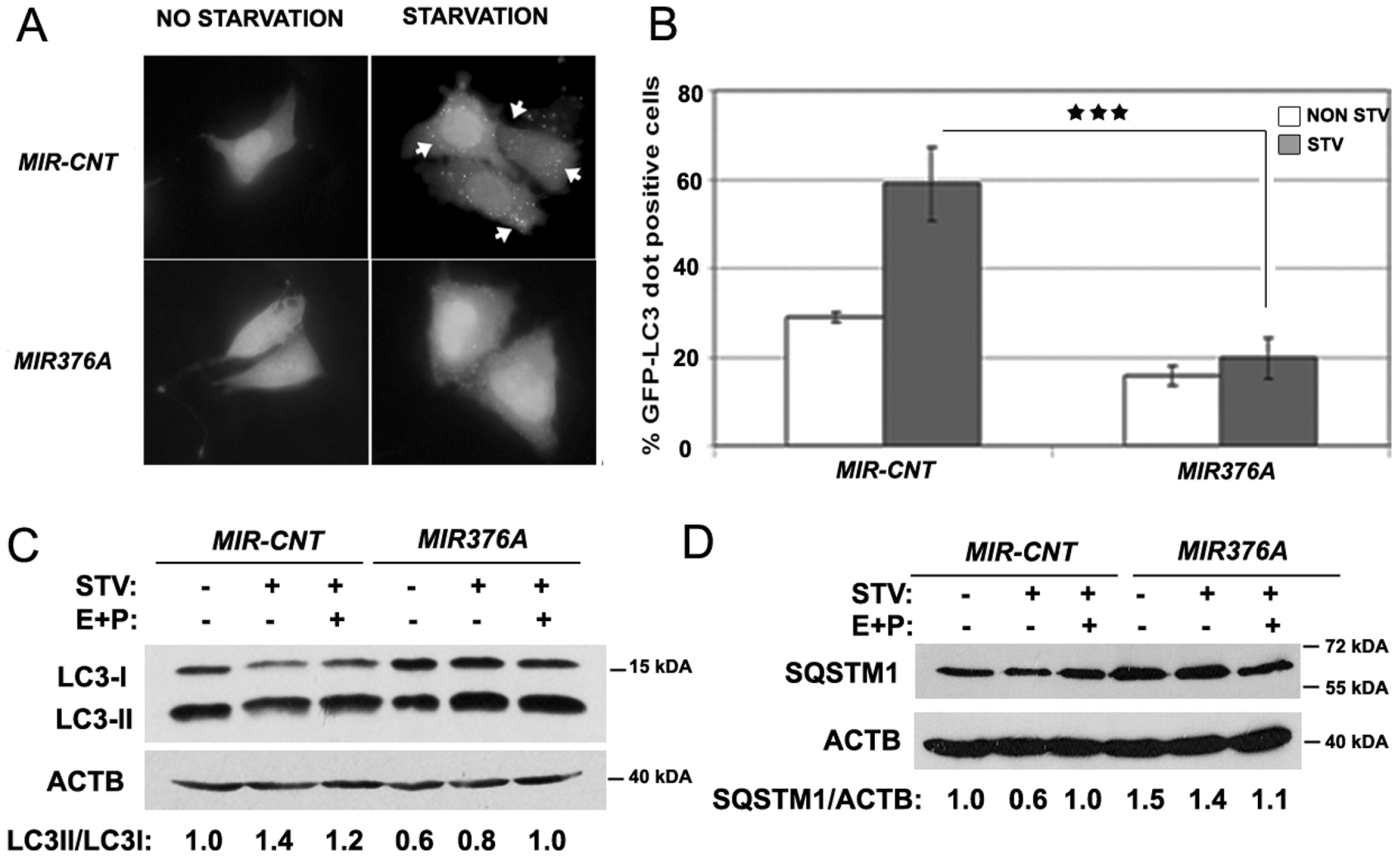
Effect of *MIR376A* overexpression on autophagy. (A) MCF-7 cells were co-transfected with either *MIR-CNT* (control plasmid) or *MIR376A* and GFP-LC3 plasmid, and GFP-LC3 dot formation was analyzed. White arrows indicate clusters of the GFP-LC3 dots in cells. (B) Quantification of the experiments in A. *MIR376A* overexpression, but not *MIR-CNT* expression, blocked starvation (STV)-induced autophagy (mean ± S.D. of independent experiments, n = 4, ***p<0,01). NON STV, non-starved (C) Overexpression of *MIR376A* resulted in a decrease in the autophagic activity of MCF-7 cells. Starvation-induced conversion of LC3-I to LC3-II in MCF-7 cells was analyzed. Tests were performed in the presence or absence of E64d (10 µg/ml) and Pepstatin A (10 µg/ml) (E+P). LC3-II/LC3-I densitometric ratios were marked. ACTB was used as a loading control. (D) *MIR376A* blocked starvation induced SQSTM1 degradation in MCF-7 cells. ACTB was used as a loading control. SQSTM1/ACTIN densitometric ratios were marked.

Firstly, we transiently overexpressed *MIR376A1* (*MIR376A* herein) together with the autophagy marker GFP-LC3 in MCF-7 breast cancer cells. Detection of cytoplasmic puncta formation by otherwise soluble GFP-fused MAP1LC3 (shortly LC3) protein is a commonly used method to follow autophagy activation using microscopy. As shown in Figure 2A and B, overexpressed *MIR376A* could repress starvation-induced GFP-LC3 puncta formation. To confirm these results with a complementary technique, we analyzed the effect of *MIR376A* overexpression on the conversion of endogenous free LC3 protein (LC3-I) to its lipidated and autophagic vesicle-associated form (LC3-II). As seen in Figure 2C, LC3-I conversion to LC3-II was decreased in miRNA transfected MCF-7 cells compared to controls.

Autophagic cargo receptor protein p62/SQSTM1 is also carried by autophagosomes to lysosomes and degraded there during the process.[18] Indeed, starvation resulted in SQSTM1 degradation in cells transfected with the control miRNAs, and inhibition of the lysosomal enzymes blocked its degradation (Figure 2D). Yet, in starved cells overexpressing *MIR376A*, SQSTM1 was neither degraded following starvation, nor accumulated after lysosomal inhibition, confirming autophagy blocking activity of the miRNA.

**Figure 3:**
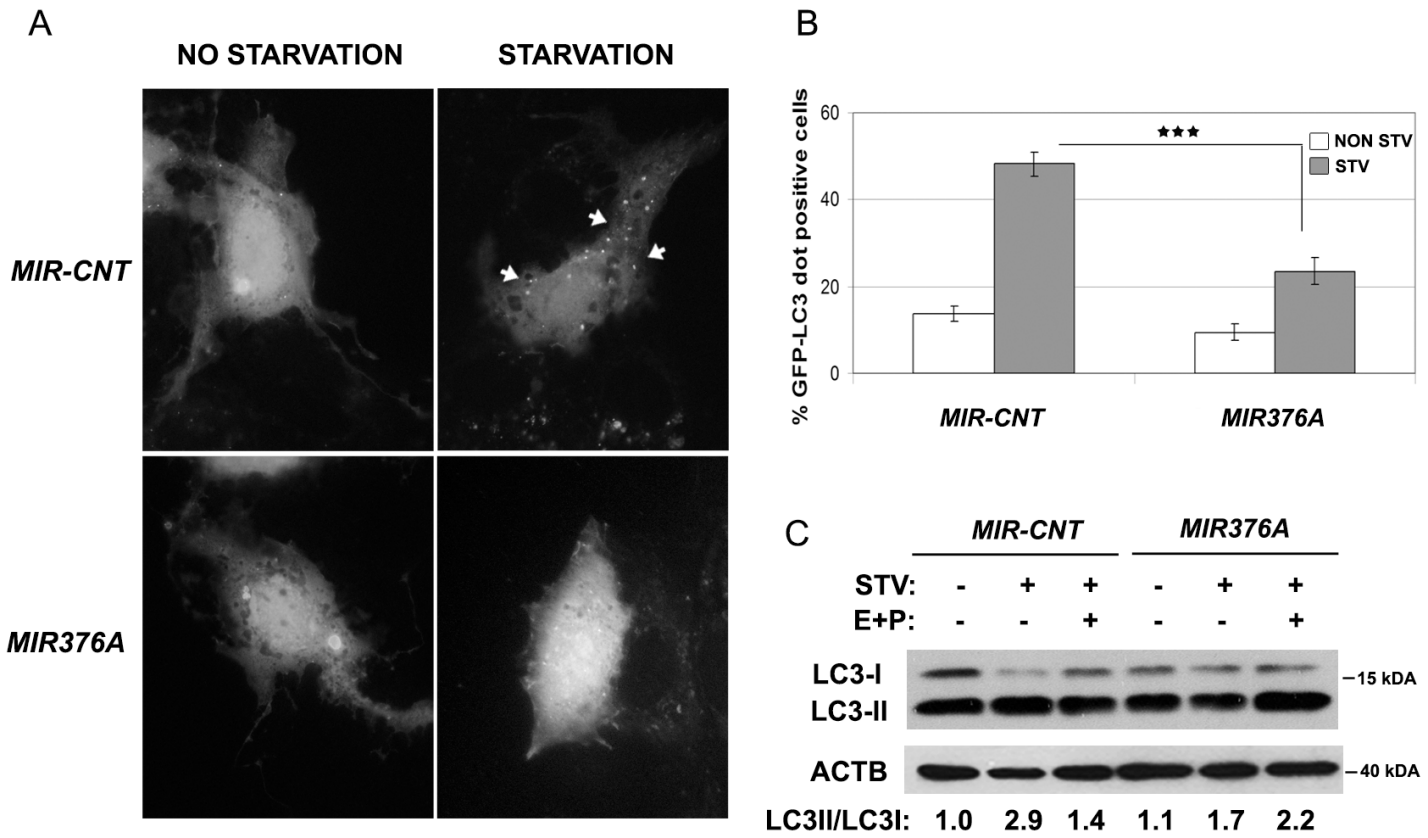
*MIR376A* overexpression blocked autophagy in Huh-7 cells. (A) *MIR376A* blocked GFP-LC3 dot formation under starvation condition. (B) Quantitative analysis of experiments in A (mean ± S.D. of independent experiments, n = 3, ***p<0,01). (C) Overexpression of *MIR376A* resulted in decreased autophagic flux in Huh-7 cells. Starvation-induced conversion of LC3-I to LC3-II was analyzed. Tests were performed in the presence or absence of E64d (10 µg/ml) and Pepstatin A (10 µg/ml) (E+P). LC3-II/LC3-I densitometric ratios were marked. ACTB was used as a loading control.

We also performed tests in Huh7 hepatocellular carcinoma cells. Similar to the results obtained in MCF-7 cells, *MIR376A* overexpression could block starvation-induced GFP-LC3 dot formation (Figure 3A and B) and LC3 lipidation (Figure 3C) in Huh7 cells as well.

All these results showed that, *MIR376A,* another gene encoded by the miRNA gene cluster in the *DLK1/GTL2* genomic region, was a new miRNA regulator of starvation-activated autophagy.

### Autophagy-related targets of *MIR376A*

**Figure 4:**
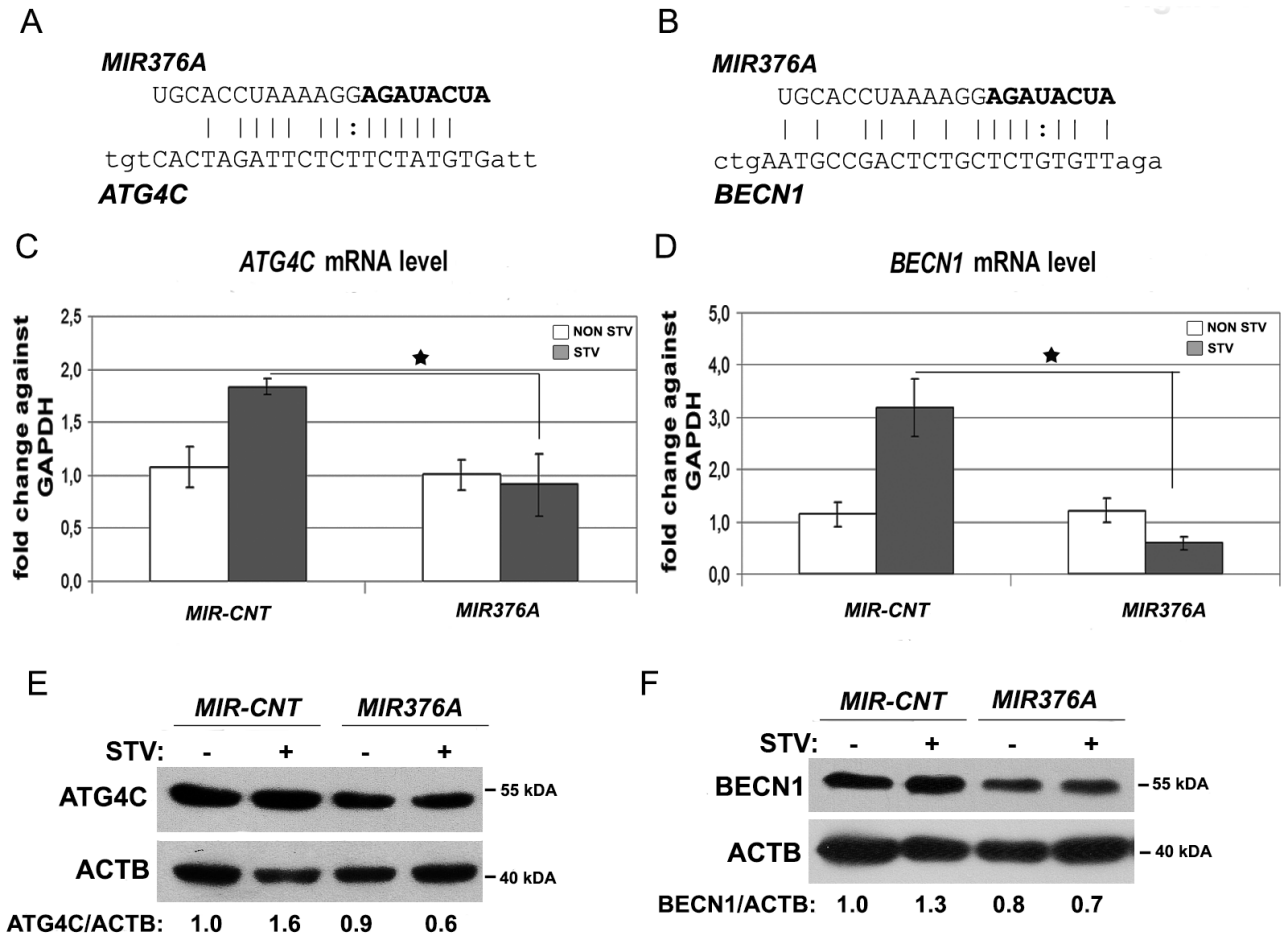
ATG4C and BECN1 were targets of *MIR376A*. (A and B) *MIR376A* mature miRNA sequence and *MIR376A* binding target sequences in the 3' UTR of *ATG4C* (A) and *BECN1*(B) mRNAs were depicted. *MIR376A* seed sequence was shown in bold letters and and complemetary sequences were indicated. (C and D) qPCR analysis of *ATG4C* and *BECN1* mRNA expression in control miRNA (*MIR-CONT*) or *MIR376A* overexpressing cells under non-starved (NON STV) or starved (STV) condition (mean ± S.D. of independent experiments, n = 3, *p<0,05). (E and F) Immunoblots showing ATG4C and BECN1 protein levels following control miRNA (*MIR-CONT*) or *MIR376A* overexpression. ACTB was used as a loading control. ATG4C/ACTB or BECN1/ACTB densitometric ratios were marked.

We have previously described *ATG4C* and *BECN1* as autophagy-related targets of *MIR376B*. Since *MIR376A* seed sequence was identical to that of *MIR376B*, it could potentially target the MRE sequences found in the 3′ UTR regions of these autophagy genes (Figure 4A and B). Indeed, mRNA levels of both genes were decreased upon *MIR376A* overexpression (Figure 4C and D). Additionally, *MIR376A* overexpression led to a decrease in the levels of both ATG4C (Figure 4E) and BECN1 (Figure 4F) proteins. Therefore, similar to *MIR376B*, *MIR376A* could also regulate expression levels of two key proteins in the autophagy pathway.

### Effect of *MIR376A* antagomirs on *ATG4C* and *BECN1* levels

**Figure 5:**
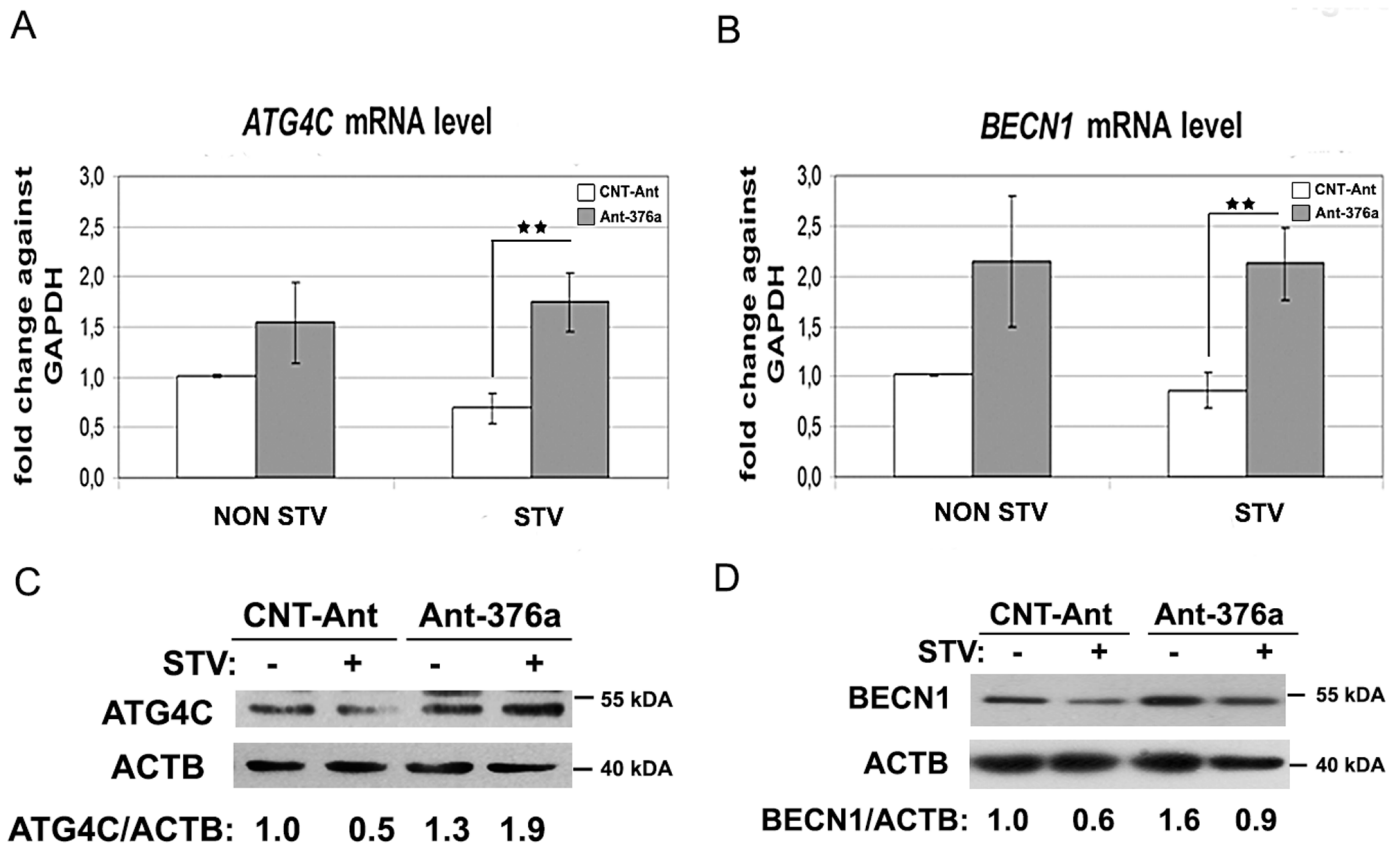
Effect of antagomirs on *MIR376A* target expression. (A and B) qPCR analysis of *ATG4C* and *BECN1* mRNA levels in MCF-7 cells transfected with control antagomirs (CNT-Ant) or antagomir-376a (Ant-376a) (mean ± S.D. of independent experiments, n = 3, **p<0,03 for *ATG4C*, and n = 3, **p<0,03 for *BECN1*). Results were expressed as fold changes against *GAPDH* mRNA levels. (C and D) ATG4C and BECN1 protein levels were increased following antagomir-376a (Ant-376a) transfection. ATG4C/ACTB or BECN1/ACTB densitometric ratios were marked.

To check whether blockage of the endogenous miRNA would affect *ATG4C* and *BECN1* expression levels, we used antagomirs (chemically engineered oligonucleotide anti-miRNAs) specifically neutralizing *MIR376A*. While control antagomirs (CNT-Ant) showed no significant effect, introduction of *MIR376A*-specific antagomirs (Ant-376A) into cells led to a significant increase in *ATG4C* mRNA (Figure 5A) and protein levels (Figure 5C). Similar antagomir-related changes were observed in *BECN1* mRNA (Figure 5B) and protein (Figure 5D) levels. Therefore, endogenously expressed cellular *MIR376A* also played a role in the suppression of autophagy-related gene expression.

### *MIR376A* directly targeted *ATG4C* and *BECN1* 3′ UTR sequences

**Figure 6:**
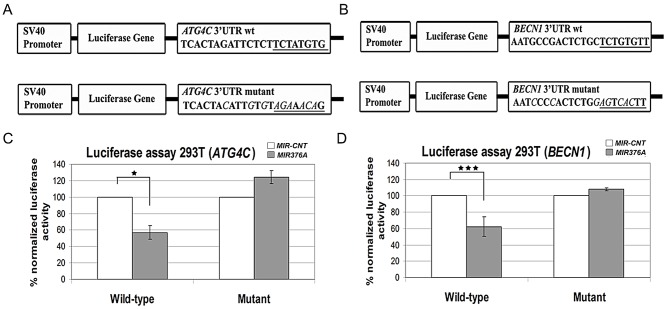
*MIR376A* controlled miRNA-response elements (MREs) in *ATG4C* and *BECN1* mRNA 3′ UTRs. Schemes showing *ATG4C* (A) and (B) *BECN1* MRE (top), or their mutated versions (bottom) placed in the 3' UTR of the luciferase gene in the pGL3 vector. Mutated residues were *italicized*. *MIR376A* seed target sequence was underlined. (C and D) Normalized luciferase activity measuremets of lysates from 293T cells that were co-transfected with either *MIR-CNT* or *MIR376A* plasmids together with the wild-type or mutant luciferase constructs (mean ± S.D. of independent experiments, n = 3 for *ATG4C* tests, *p<0,05 and n = 5 for *BECN1* tests, ***p<0,01).

To prove that negative regulation of the autophagy proteins by *MIR376A* was a result of a direct effect of the miRNA, reporter luciferase vectors containing predicted 3′ UTR MRE sequences in *ATG4C* and *BECN1* mRNAs were prepared. Mutant versions of these vectors were constructed as well (Figure 6A and B). 293T cells were transfected with these constructs together with *MIR-CNT* or *MIR376A,* and luciferase activities were measured. Overexpression of *MIR376A* but not *MIR-CNT* resulted in a decrease in luciferase expression from vectors containing wild-type MRE sequences (Figure 6C and D, Wild-type). Mutation of the MREs in *ATG4C* or *BECN1* sequences abolished the effect of the miRNA on luciferase expression (Figure 6C and D, Mutant). Therefore *MIR376A* controlled ATG4C and BECN1 levels by directly affecting specific MRE sequences in the 3′UTR regions of these autophagy genes.

### Role of endogenous *MIR376A* in the control of starvation-induced autophagic activity

**Figure 7:**
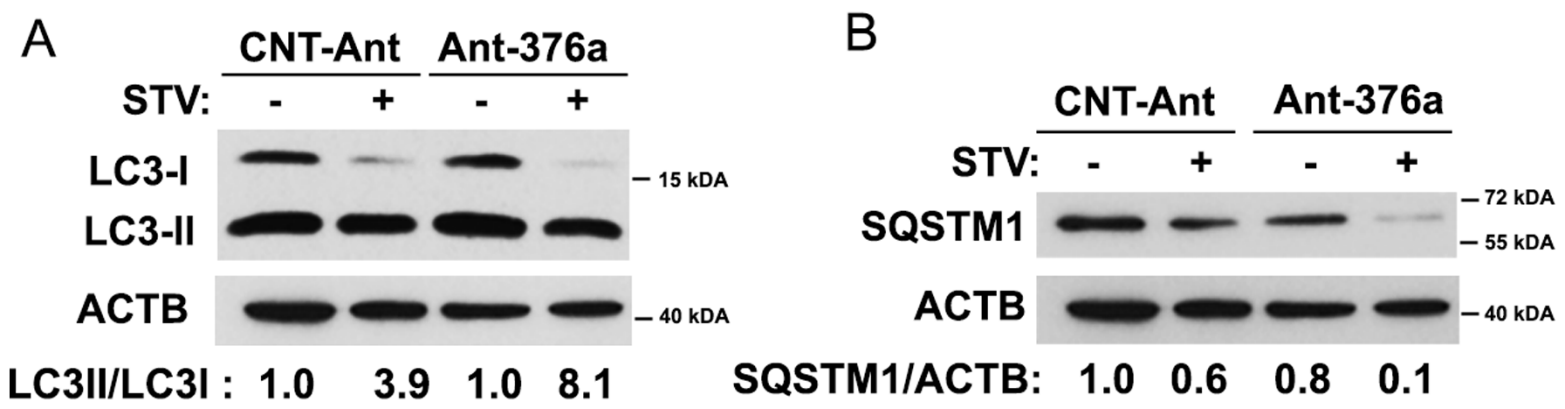
Endogenous *MIR376A* limits starvation-induced autophagy. (A) Blockage of endogenous *MIR376A* by Ant-376a, but not CNT-Ant further stimulated starvation (STV)-activated LC3-I to LC3-II conversion in MCF-7 cells. ACTB was used as a loading control. LC3-II/LC3-I densitometric ratios were marked. (B) Ant-376a, but not CNT-Ant resulted in further activation of SQSTM1 protein degradation following starvation in MCF-7 cells. SQSTM1/ACTB ratios were marked.

To further reveal the role of endogenous *MIR376A* in autophagy regulation, we transfected cells with control or *MIR376A* antagomirs, and analyzed conversion of LC3-I to LC3-II and SQSTM1 degradation as markers of the autophagic activity. Interestingly, we observed that blockage of endogenous *MIR376A* further increased LC3-II/I ratios (Figure 7A). Moreover, starvation-induced degradation of the autophagy receptor SQSTM1 was further increased in cells transfected with *MIR376A* antagomirs compared to controls (Figure 7B). These results underlined the importance of endogenous *MIR376A* activity in the control of the amplitude of starvation-induced autophagy responses of cells.

### Response of endogenous *MIR376A* and *MIR376B* levels to starvation

**Figure 8:**
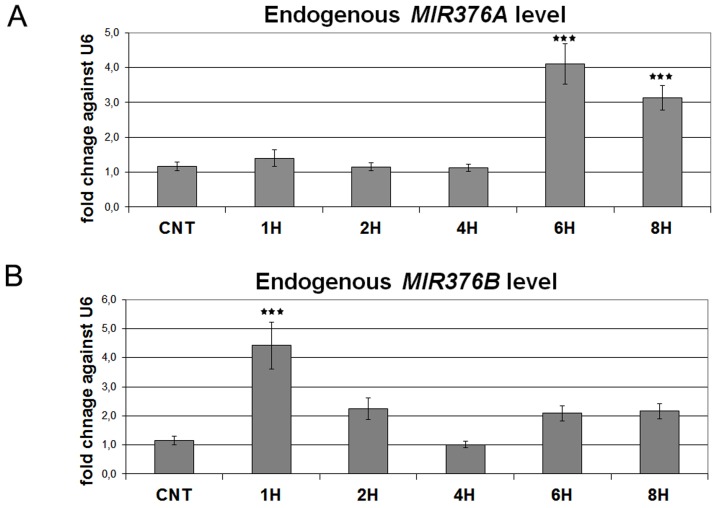
*MIR376A* and *MIR376B* level changes in response to starvation stress. (A and B) TaqMan quantitative PCR (qPCR) analysis of endogenous *MIR376A* (A) and *MIR376B* (B) levels under control (CNT, no starvation) or starvation (STV, 1 to 8 hours) conditions. TaqMan qPCR data was normalized using *U6 small nuclear 1 (RNU6-1)* (U6) mRNA levels (mean ± S.D. of independent experiments, n = 5 for both A and B, ***p<0,01).

We have previously reported that autophagy-related miRNAs accumulated following starvation or mTOR inhibition.[10], [11] To check whether endogenous *MIR376A* and *MIR376B* levels were similarly increased during starvation stress, we performed a kinetic analysis using TaqMan qPCR. Although cellular levels of *MIR376A* and *MIR376B* were increased in cells exposed to starvation stress, kinetics of the upregulation was different between individual miRNAs. In starved cells, while endogenous *MIR376A* reached peak levels following a 6 hours lag period (Figure 8A), endogenous *MIR376B* levels rapidly increased after 1 hr of stress, but the response was transient (Figure 8B). Therefore, the two miRNAs responded to autophagy-inducing starvation signals with different kinetics.

## Discussion

In this study, we demonstrated that another miRNA encoded by the *DLK1/GTL2* region and belonging to the *MIR376* family, namely *MIR376A* was an autophagy regulatory miRNA. Inhibitory effect of *MIR376A* on autophagy was demonstrated using various autophagic tests including GFP-LC3 puncta analysis, LC3II/LC3I gel shift and p62/SQSTM1 degradation tests. The miRNA affected ATG4C and BECN1 protein levels as well as the mRNA levels. *MIR376A* directly and specifically affected the mRNAs of the autophagy genes, since MREs in the 3′ UTR of both *ATG4C* and *BECN1* were responsive to the inhibitory effects of the miRNA in luciferase tests, and mutations in the relevant regions abolished the suppressive effects. Contribution of the endogenous *MIR376A* to the control of basal and stress-activated autophagy was documented as well. Specific antagomirs against the miRNA led to a clear increase in both ATG4C and BECN1 mRNA and protein levels, and stimulated autophagic activity in cells.

Since ATG4C and BECN1 function at two different steps of autophagic vesicle formation, targeting of both of these key autophagy proteins at once by the *MIR376* family of miRNAs allows a robust decrease in the autophagic activity. In fact, while BECN1 controls the production of PI3-P and regulates of autophagic vesicle nucleation sites, ATG4 is important for LC3 lipidation and in autophagic vesicle membrane elongation. In line with the importance of these proteins in autophagy control, both proteins were reported to be regulated by diverse signals and pathways. In fact, BECN1 was subject to regulation by transcriptional upregulation, protein-protein interactions, phosphorylation and ubiquitination.[19], [20], [21] ATG4 was also reported to be regulated in various ways including transcriptional upregulation and reactive oxygen species [22] Moreover, miRNAs other than *MIR376* family were shown to target BECN1 or ATG4 as well. For example, miR-30a targeted BECN1 3′ UTR but at a different and unrelated MRE. Similarly, ATG4 was targeted by autophagy-related *MIR101*.[23]

Although *MIR376A and MIR376B* genes are separated from each other by only hundreds of bases and their products are not identical but closely-related (Figure 1B and 1C). Hence, their cellular functions could be overlapping. Indeed, we have previously shown that *MIR376B* was also blocking starvation-induced autophagy through its effects on ATG4C and BECN1 levels. Yet, we observed in this study that kinetics of miRNA responses during starvation stress were divergent. While endogenous levels of *MIR376A* increased after 6 hours of starvation, *MIR376B* levels increased following a short starvation period, but declined rapidly. The observed difference in the response of *MIR376A* and *MIR376B* might be relevant to miRNA function during stress. Results presented here provide evidence that, the physiological function of these miRNAs is to control and limit the amplitude of autophagic activity which is strongly stimulated by starvation stress. A sequential accumulation of individual miRNAs might allow a sustained and a two-step control of the autophagic activity depending on the duration of stress. Since uncontrolled autophagic activity could be detrimental for cells, and under certain conditions lead to cell death [24] restriction of aberrant activation of autophagy by miRNAs under stressful conditions might prevent cellular demise and give cells a chance to recover a transient stress.

Data presented here point out to the presence of distinct control mechanisms resulting in dissimilar *MIR376A* and *MIR376B* response kinetics. Transcriptional and/or post-transcriptional regulatory mechanisms might be in the origin of these observations. According to Kawahara et.al (2007), miR-376 family members were transcribed as a long primary transcript.[17] Reports by Seitz et al. and Teferedegne et al. confirmed experimentally the presence of a common transcript including *MIR376A and MIR376B.* [16], [25] In line with these data, a recent study showed that the expression of *MIR376A, MIR376B and MIR376C* was regulated by a transcription factor called AIRE in a coordinated manner.[26] Although experimental confirmation is currently missing, our bioinformatics analyses revealed that predicted short promoter regions of these two miRNAs could be responsive to overlapping but different transcription factors, indicating that single miRNA transcripts could also co-exist (unpublished data by Ilknur Melis Durasi, Osman Ugur Sezerman and Devrim Gozuacik). If the dominant transcript is a long and polycistronic mRNA coding for both *MIR376A and MIR376B,* observed differences in the levels and stress-related kinetics of these miRNAs might be the result of posttranscriptional regulation. Indeed, posttranscriptional events were previously shown to affect stability and function of some miRNAs. For example, regulation by DICER was reported to affect the levels of let-7.[27] Moreover, complexes Ago proteins were shown to determine the stability of various miRNAs.[28] Further studies are required to reveal the nature and molecular details of posttranscriptional events that are responsible for the differential regulation of *MIR376A* and *MIR376B* levels under stress-inducing conditions.

Differential editing of *MIR376* family members was reported in a number of tissues.[17] For example, while +44 site in the seed sequence of *MIR376A* was not edited in the mouse cortex, in the same tissue *MIR376B* was highly edited (around 50–60%) at this position.[17] Since editing in the seed region was shown to change target specificity of miRNAs including MIR376A* [17], [29] in addition to relative ratios of *MIR376A* and *MIR376B,* ratios between edited versus non-edited versions of *MIR376* family members might be important factors leading to changes in autophagy levels in different tissues and cells. Additionally, *MIR376A* might be compensating autophagy-related functions of *MIR376B* in tissues where the latter was predominantly edited and, vice versa. Overall, editing frequencies of *MIR376* family members and their relative ratios might be critical factors determining the kinetics and the amplitude of autophagic responses in individual tissue and cell types. Further studies are required to analyze and integrate these observations to cellular and organismal autophagy responses.

Tissue-specific expression differences were observed between *MIR376A* and *MIR376B* under physiological conditions. While *MIR376B* is highly expressed in the spleen and adrenal gland, *MIR376A* was reported to be abundant in the brain and uterus (Fig. S1).[30] A number of studies in the literature, showed the importance of *MIR376A* during development and differentiation-related events including erythroid differentiation [31], keratinocyte differentiation [32] and skeletal muscle development.[33] *MIR376A* was also shown to be upregulated during chemical and replicative senescence in human fibroblasts.[34]

Additionally, changes in *MIR376* levels were observed under pathological conditions. While *MIR376A* was downregulated in esophageal cancer [35] and melanomas [36] and upregulated in salivary gland adenomas,[37] and pancreatic carcinomas,[38], [39] *MIR376B* was differentially expressed in uterine leiomyomas [40] and renal cell carcinomas.[41] Moreover, changes in *MIR376A* levels could be used as a marker to distinguish acute myeloid leukemia subtypes, but *MIR376B* could serve as a breast cancer subtype marker.[42] Additionally, *MIR376A* and *MIR376B* were subjected to variable levels of editing in glioblastomas.[43]

Autophagy serves as a cellular defense mechanism against various pathogens including viruses.[44], [45] Not surprisingly, as a countermeasure, viruses evolved ways of modulating autophagy during productive and latent stages of the viral infection.[46] miRNAs of both viral and cellular origins were shown to affect cellular responses to viruses such as apoptosis, therefore, it is possible that autophagy-regulating miRNAs play a role in the battle between viruses and the host. In line with a possible role of *MIR376* family in virus-host interactions, a miRNA profiling revealed differential expression of *MIR376A and MIR376B* in control versus HIV-1 positive peripheral blood mononuclear cells.[47] In another study, an interesting interplay between viral miRNAs and *MIR376A* was reported.[48] MREs for *MIR376A* in the 3′ UTR of the MICB mRNA (a stress-induced ligand necessary for host cell recognition and destruction by natural killer cells) overlapped with the MRE of Kaposi’s sarcoma-associated herpesvirus (KSHV) miRNA, miR-K12-7. The *MIR376A* MRE was also in the vicinity of that of miR-UL112 of human cytomegalo virus (HCMV). Surprisingly, when *MIR376A* was used in combination with KSHV or HCMV miRNA, no antagonism but an increase in target MICB downregulation was observed. In the light of our data, here contribution of *MIR376A* to viral infection might not be limited to the attenuation of immune responses through MICB downregulation, but it might also involve blockage of antiviral autophagic degradation, autophagy-related antigen presentation on MHC molecules and perhaps autophagic cell death. Indeed, KSHV was previously shown to downregulate autophagy, apoptosis and cell death using its viral FLIP and BCL-2 proteins that target autophagy-related proteins ATG3 and BECN1, respectively. [49], [50] *MIR376A* and possibly other autophagy-regulating miRNAs might be usurped by viruses to overcome the antiviral effects of autophagy.

Altogether, our results underline the importance of *MIR376A* and *MIR376B,* and miRNAs in general, in the control of autophagic responses of cells and tissues. miRNA-mediated regulation provides a flexible and dynamic mechanism for the regulation of autophagy under various stress conditions, and adds another layer of regulation for critical cell death and survival decisions in health and disease.

## Materials and Methods

### Plasmid constructs

The pMSCV-blast-miR plasmids, containing either hsa-miR-376a1 human miRNA or control miRNA (hTR-human telomerase RNA), were constructed as described previously.[51] For luciferase tests, miRNA response elements (MRE) located in the 3′UTR of *BECN1* or *ATG4C* genes (*ATG4C* Genbank accession number: AK027773, bases 2383-2403, and *BECN1* Genbank accession number: NM_003766, bases 2030-2051), or their mutant versions were cloned into the 3′UTR region of the luciferase gene in the pGL3 vector (Promega, E1741) as previously described.[10] *ATG4C* plasmid was purchased from Origene (SC126496). *BECN1* cDNA ORF was cloned into the pcDNA3 mammalian expression plasmid using RT-PCR.

### Cell culture and transfection

Dulbecco’s modified Eagle’s medium (DMEM; Sigma, D5671) supplemented with 10% (v/v) fetal bovine serum (FBS; Biochrom KG, S0115) and antibiotics (Penicillin/Streptomycin; Biological Industries, 03-031-1B) were used to culture MCF-7 human mammary carcinoma cells, Huh7 human hepatocellular carcinoma cells and 293T human embryonic kidney cells in a 5% CO_2_-humidified incubator at 37°C. Cells were cultured in Earl’s balanced salt solution (EBSS; Biological Industries, BI02-010-1A) to activate starvation-induced autophagy. For autophagic flux analyses, cells were cultured in the absence or presence of lysosomal protease inhibitors E64d (10 μg/ml) (Santa Cruz, SC201280A) and pepstatin A (10 μg/ml) (Sigma, P5318).

Polyethyleneimine (PEI; PolySciences Inc., 23966) transfection method was used to transiently transfect MCF-7 and Huh7 cells.[52] For transfection of 293T cells, calcium phosphate co-precipitation method was used according to standard protocols.

### Quantitative GFP-LC3 analyses

48h post-transfection, GFP-LC3 dot positivity was quantified following 2 hours (MCF-7 cells) or 4 hours (Huh7 cells) starvation in the EBSS medium. 10 or 15 GFP-LC3 dots per cell were considered as a threshold for the basal autophagic activity in MCF-7 and Huh7 cells, respectively. Minimum 150 GFP positive cells were counted under each condition, and percentage of GFP-LC3 positivity was expressed as a percentage of GFP-LC3 dot positive cells within the total transfected cell population.

### miRNA target prediction

Bioinformatics tools, Microcosm Targets (http://www.ebi.ac.uk/enright-srv/microcosm/cgi-bin/targets/v5/search.pl), and MiRanda (http://www.microrna.org/microrna/home.do) were utilized to determine miRNA potential target mRNAs.

### Immunoblotting and antibodies

Protein extracts from cells were prepared and immunoblotted as previously described [53] using antibodies specific to BECN1 (Santa Cruz, sc-11427), LC3B (Novus, NB100-2331), ATG4C (Sigma-Aldrich, AB75056), SQSTM1 (Abnova, H00008878) and ACTB (Sigma-Aldrich, A5441). ImageJ software was used to quantify protein band intensities.[54]

### RNA isolation, RT-PCR analysis and Real-time RT-PCR

Total RNA was extracted using TRIzol reagent (Sigma-Aldrich, T9424) according to the manufacturer’s instructions. SYBR® Green Quantitative RT-PCR kit (Roche, 04-913-914-001) was utilized for single step qRT-PCR reactions. The 2^−▵▵CT^ method was applied for the quantification of mRNA changes, and *GAPDH* (Glyceraldehyde-3-phosphate dehydrogenase) mRNA was used as control. Primers used in the study were: *BECN1* primers 5′-AGGTTGAGAAAGGCGAGACA-3′; 5′-GCTTTTGTCCACTGCTCCTC-3′; *ATG4C* primers 5′-GCATAAAGGATTTCCCTCTTGA-3′; 5′-GCTGGGATCCATTTTTCG-3′, and *GAPDH* primers 5′-AGCCACATCGCTCAGACAC-3′; 5′-GCCCAATACGACCAAATCC-3′. Reactions were performed in duplicates and independent experiment repeat numbers (n) were marked.

### Endogenous miRNA quantification by TaqMan RT-qPCR

FastStart Universal Probe Master kit (ROCHE, 04913957001) and iCycler iQ thermal cycler (BioRad) was used for TaqMan qPCR reactions. Reactions were previously described.[11] Primers used in this study were: Stemloop primer for *MIR376A*: 5′-GTCGTATCCAGTGCAGGGTCCGAGGTATTCGCACTGGATACGACACGTGGATTTTCCTCTATGAT-3′; Forward primer for *MIR376A*: 5′-ATTAATCATAGAGGAAATCCACG-3′; Reverse primer for *MIR376A:* 5′-GTGCAGGGTCCGAGGT-3′; Probe for *MIR376A*: 5'(6-FAM)- TGCACTGGATACGACACGTGGA-(TAMRA-Sp)3'. Primers for U6 small nuclear 1, and *MIR376B* were previously described.[10]

### Dual luciferase reporter assay

Firefly and renilla activities in cell extracts were measured using dual-luciferase reporter assay system (Promega, E1910) according to manufacturer’s instructions. Results were expressed as firely luciferase activity normalized to renilla luciferase activity and analyzed as described previously.[10], [11]

### Antagomir tests

miRIDIAN® microRNA Hairpin Inhibitors (antagomirs) against hsa-miR-376a1 (IH-300683-05-0005) and a control antagomir (miRIDIAN microRNA Hairpin Inhibitor Negative Control (IN-001005-01-05)) were purchased from Dharmacon. Antagomir transfections (200 nM) were performed using the PEI transfection method.[52]

### Statistical analyses

Statistical analyses were performed using Student’s two-tailed t-test. Data were represented as means of ± SD of n independent experiments. Values of p<0.05 were considered as significant.

## Supporting Information

Figure S1 **Data extracted and analyzed using miRNA body map online website (** http://www.mirnabodymap.org/ **) based on the high throughput microRNA expression analysis in normal tissues.** [**30**]
(TIF)
