# Correction: *MIR376A* Is a Regulator of Starvation-Induced Autophagy

**DOI:** 10.1371/journal.pone.0103385

**Published:** 2014-07-17

**Authors:** The PLOS ONE Staff

There is a missing grant number in the Financial Disclosure (FD) statement of this article. Please refer to the correct FD statement below:

This work was supported by The Scientific and Technological Research Council of Turkey (TUBITAK) 1001 Grant number: 112T272 and Sabanci University. D.G. and A.K are recipients the Turkish Academy of Sciences (TUBA) GEBIP Award. D.G. is a recipient of the EMBO Strategical Development and Installation Grant (EMBO-SDIG) and A.K. is a recipient of the TUBITAK Incentive Award. G.K. and K.A.T. are recipients of Yousef Jameel and TUBITAK-BIDEB PhD Scholarships, respectively. The funders had no role in study design, data collection and analysis, decision to publish, or preparation of the manuscript.
