# Supplementary figures and images for: MIR376A Is a Regulator of Starvation-Induced Autophagy
Source: PLoS One. 2013 Dec 16;8(12):e82556. doi: 10.1371/journal.pone.0082556 (PMC3864973; doi:10.1371/journal.pone.0082556)

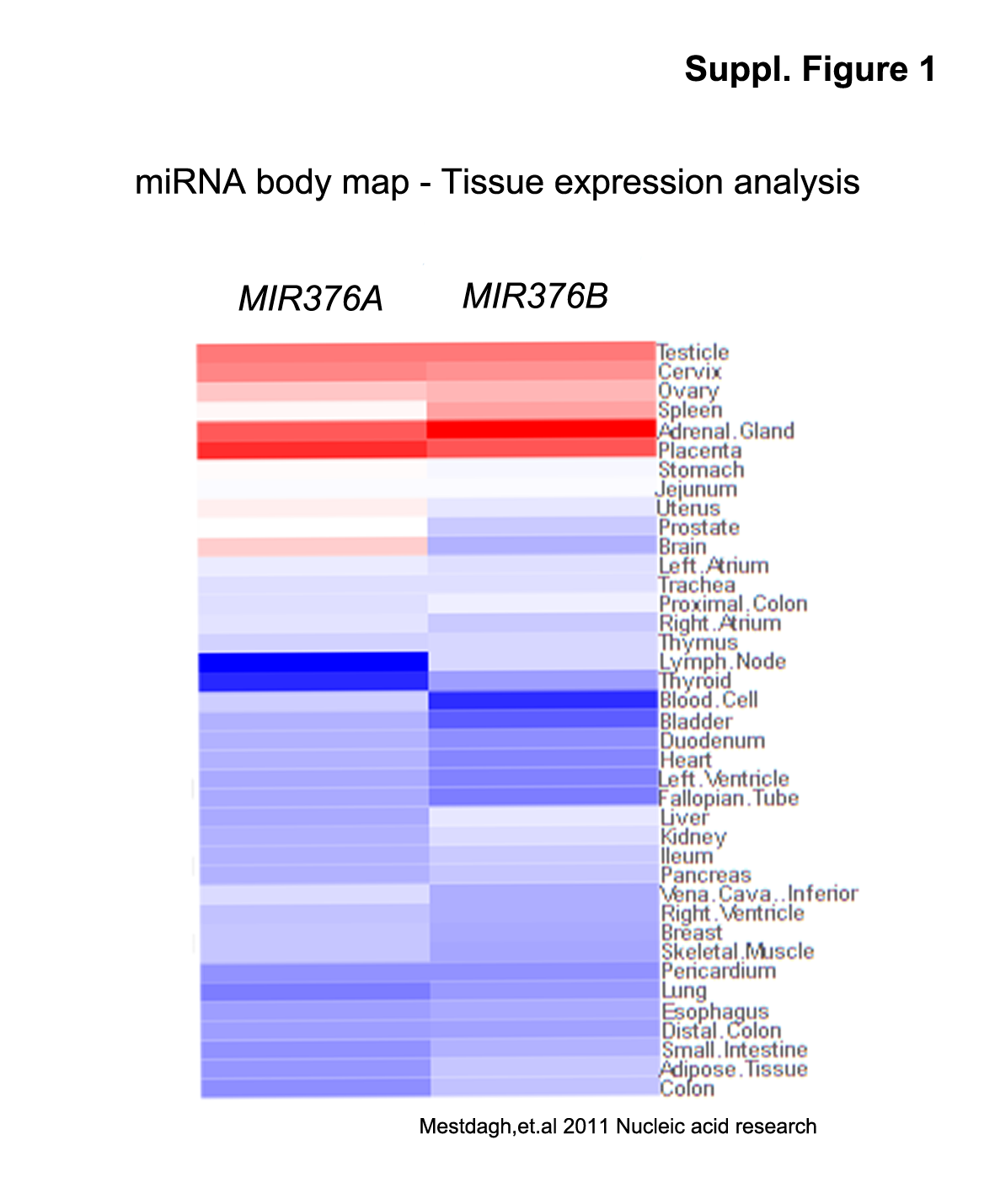

Supplement: Figure S1 — Data extracted and analyzed using miRNA body map online website ( http://www.mirnabodymap.org/ ) based on the high throughput microRNA expression analysis in normal tissues. [30] (TIF) [file pone.0082556.s001.tif]
